# New Functional Organic Materials and Their Photoelectric Applications: A New Open Special Issue of *Materials*

**DOI:** 10.3390/ma15103444

**Published:** 2022-05-11

**Authors:** Ruonan Yin, Jing-Jing Lv

**Affiliations:** Key Laboratory of Carbon Materials of Zhejiang Province, Institute of New Materials and Industrial Technologies, Wenzhou University, Wenzhou 325035, China; yin02176914@163.com

*New Functional Organic Materials and Their Photoelectric Applications* is a new open Special Issue of *Materials*, which focuses on designing and fabricating advanced functional organic optoelectronic materials and makes great contributions to investigating their properties, related applications, and underlying mechanisms.

Organic optoelectronic materials refer to organic materials used in optoelectronic technology, which have the characteristics of generation, conversion and transmission of photons and electrons. Due to the characteristics of large-scale manufacture, flexible substrate, environmental friendliness, and portable size, organic photoelectric materials are widely used in organic light-emitting diodes, organic transistors, organic solar cells, and other applications.

For instance, the photosensitive organic materials are extensively applied in organic solar cells (OSCs) to transform solar power into electricity [1]. The advanced OSCs have the outstanding advantages like low cost, light weight, flexibility, simple manufacturing process, and roll-to-roll fabrication, which are expected to be employed in power watches, portable calculators, toys, flexible crimpable systems and other systems in the future.

The research interest of the section *New Functional Organic Materials and Their Photoelectric Applications* not only includes the design and fabrication of organic optoelectronic materials, but also studies the applications of organic optoelectronic materials in other fields and further explores the materials in practical optoelectronic devices.
